# Obesogenic home food availability, diet, and BMI in Pakistani and White toddlers

**DOI:** 10.1111/mcn.13138

**Published:** 2021-01-19

**Authors:** Madison N. LeCroy, Maria Bryant, Sandra S. Albrecht, Anna Maria Siega‐Riz, Dianne S. Ward, Jianwen Cai, June Stevens

**Affiliations:** ^1^ Department of Nutrition, Gillings School of Global Public Health University of North Carolina at Chapel Hill Chapel Hill North Carolina USA; ^2^ Department of Epidemiology and Population Health Albert Einstein College of Medicine New York New York USA; ^3^ National Institute for Health Research Career Development Fellow, Clinical Trials Research Unit, Leeds Institute of Clinical Trials Research University of Leeds Leeds UK; ^4^ Carolina Population Center University of North Carolina at Chapel Hill Chapel Hill North Carolina USA; ^5^ Department of Epidemiology, Mailman School of Public Health Columbia University New York New York USA; ^6^ School of Nursing University of Virginia Charlottesville Virginia USA; ^7^ Departments of Nutrition and Biostatistics and Epidemiology, School of Public Health and Health Sciences University of Massachusetts Amherst Amherst Massachusetts USA; ^8^ Department of Biostatistics, Gillings School of Global Public Health University of North Carolina at Chapel Hill Chapel Hill North Carolina USA; ^9^ Departments of Nutrition and Epidemiology, Gillings School of Global Public Health University of North Carolina at Chapel Hill Chapel Hill North Carolina USA

**Keywords:** Asian, child, diet, infant, obesity, preschool, snack food, sugar‐sweetened beverages

## Abstract

Individuals of South Asian ethnicity have an increased risk for obesity and related diseases. Foods available in the home during the first 1000 days (conception to 24 months old) are an important determinant of diet, yet no study has examined the association of early‐life home food availability (HFA) with later diet and obesity risk in South Asian households. We examined whether obesogenic HFA at 18 months of age is associated with dietary intake and body mass index (BMI) at 36 months of age in low‐income Pakistani and White households in the United Kingdom. In this prospective birth cohort study (Born in Bradford 1000), follow‐up assessments occurred at 18 (*n* = 1032) and 36 (*n* = 986) months of age. Variety and quantity of snack foods and sugar‐sweetened beverages (SSBs) in the home and consumed were measured using the HFA Inventory Checklist and food frequency questionnaires, respectively. BMI was calculated using measured length/height and weight. Multinomial logistic regression models examined associations between HFA and tertiles of dietary intake, and multivariable linear regression models assessed associations between HFA and BMI. Pakistani households had a greater variety and quantity of snack foods and SSBs available compared with White households. Variety and quantity of snack foods and SSBs in the home at 18 months were positively associated with children's intake of these items at 36 months, but associations between HFA and BMI were null. Reducing obesogenic HFA during the first 1000 days may promote the development of more healthful diets, though this may not be associated with lower obesity risk during toddlerhood.

**Key messages**

- In this prospective cohort of 882 low‐income families in Born in Bradford 1000 (a birth cohort study in Bradford, UK), Pakistani households had a greater variety and quantity of snack foods and sugar‐sweetened beverages (SSBs) in the home compared with White households.
- Availability (variety and quantity) of snack foods and SSBs in the home at 18 months of age was positively associated with children's dietary intake of snack foods and SSBs at 36 months of age, respectively.
- We did not find an association between the availability of snack foods and SSBs in the home and BMI.

## 1 INTRODUCTION

Individuals from South Asia are one of the largest immigrant groups in the United States (US Census Bureau, 2016), Canada (National Household Survey, 2013) and the United Kingdom (UK Office for National Statistics, 2012b), with Pakistani immigrants constituting the second largest South Asian immigrant group in each country and the third largest ethnic group in the United Kingdom (UK Office for National Statistics, 2012a). Individuals of Pakistani origin are at a higher risk for obesity‐related diseases compared with Whites of the same body mass index (BMI), in part due to their increased percent body fat at a given BMI (WHO Expert Consultation, 2004). When BMI values are adjusted to represent the same percent body fat for each ethnic group (Eyre, Duncan, & Nevill, 2017; Hudda et al., 2017), Pakistani children are shown to have a significantly greater BMI compared with White children (e.g. mean BMI of 19.9 vs.18.6 kg/m^2^ for males and 19.7 vs. 19.0 kg/m^2^ for females 10–11 years of age) (Hudda et al., 2018). Thus, there is a need to identify potential targets for reducing risk for obesity in Pakistani immigrant children.

The first 1000 days (conception through 24 months of age) is a fundamental time to address the determinants of childhood obesity (Blake‐Lamb et al., 2016; Woo Baidal et al., 2016). It is during this time that food preferences and dietary habits are established (Anzman, Rollins, & Birch, 2010), with toddlers developing an affiliation for the foods they become most familiar with (Birch, Birch, Marlin, & Kramer, 1982; Rheingold, 1985). At least three quarters of toddlers' total daily energy intake comes from foods available in the home (foods prepared in home/at the grocery store) (Poti & Popkin, 2011); thus, it is not surprising that they tend to prefer the foods and beverages readily available in the household (Birch & Marlin, 1982). Existing research on home food availability (HFA) and dietary intake in toddlers and young children has largely focused on fruits and vegetables and has found a positive association between their HFA and intake (Bryant et al., 2011; Spurrier, Magarey, Golley, Curnow, & Sawyer, 2008; Wyse, Campbell, Nathan, & Wolfenden, 2011). However, the association between fruit and vegetable intake and obesity is weak, potentially due to the low consumption and low variability in intake of these food groups (Ledoux, Hingle, & Baranowski, 2011). It may be more promising to focus on the HFA of obesogenic items, such as snack foods (i.e. crisps [chips], biscuits [cookies] and sweets [candies]) and sugar‐sweetened beverages (SSBs), during the first 1000 days as a modifiable risk factor for unhealthy diets and childhood obesity (Brown, Halvorson, Cohen, Lazorick, & Skelton, 2015). Further, research on the association between the HFA of snacks and SSBs and children's dietary intake of these items is inconsistent and needs further investigation (Vaughn et al., 2016).

Few studies have characterized HFA during the first 1000 days, with only two, to our knowledge, examining the relationship between snack food and SSB HFA and diet during later childhood (Collins, Lacy, Campbell, & McNaughton, 2016; Fernando, Campbell, McNaughton, Zheng, & Lacy, 2018). However, none of these studies examined a South Asian population, and HFA has been shown to vary according to race/ethnicity (Bryant, Sahota, Santorelli, & Hill, 2015; Larson, Eisenberg, Berge, Arcan, & Neumark‐Sztainer, 2015; Ranjit, Evans, Springer, Hoelscher, & Kelder, 2015; Skala et al., 2012). Given that dietary intake during infancy and toddlerhood tracks throughout childhood (Bjelland et al., 2013; Northstone & Emmett, 2008; Park, Pan, Sherry, & Li, 2014), examining how early‐life HFA is associated with later dietary intake in South Asian households may provide key insight into a potential intervention target for childhood obesity interventions in this at‐risk population. Further, no studies of HFA during the first 1000 days have examined associations with BMI in any ethnic group.

Only one previous exploratory study conducted in a small subset (*n* = 97) of Pakistani and White homes of 18‐month‐olds from Born in Bradford 1000 (BiB1000) has examined HFA in South Asian households, finding a greater quantity of SSBs in Pakistani compared with White households (Bryant, Sahota, et al., 2015). However, the study was not powered to examine differences in HFA according to acculturation, which has been associated with HFA of SSBs in households of Hispanic/Latino adolescents (Santiago‐Torres et al., 2016).

This manuscript addresses these gaps using data from BiB1000, a multi‐ethnic birth cohort of low‐income households in the United Kingdom designed to consider exposures to obesity in early life (Bryant et al., 2013). Here, the objectives were to describe the availability of obesogenic items (snack foods and SSBs) in the homes of second‐ and third‐generation Pakistani toddlers and White toddlers and to determine if HFA of obesogenic items at 18 months of age was associated with toddler's dietary intake of snack foods and SSBs and BMI at 36 months of age. Cross‐sectional analyses were also conducted at 18 and 36 months of age to assess if HFA of these items was associated with diet and BMI in the expected directions. It was hypothesized that Pakistani homes would have more snack foods and SSBs compared with White homes, with households of third‐generation Pakistani immigrant toddlers having greater availability of these items than households of second‐generation Pakistani immigrant toddlers. Further, HFA of snack foods and SSBs was expected to be positively associated with dietary intake of these foods/beverages and BMI in both Pakistani and White toddlers.

## 2 METHODS

### 2.1 Study population

BiB1000 is a birth cohort study nested within the larger Born in Bradford (BiB) study. Detailed information on study protocols for BiB and BiB1000 are published elsewhere (Bryant et al., 2013; Raynor & Born in Bradford Collaborative Group, 2008; Wright et al., 2013). BiB is a multi‐ethnic cohort study that examines environmental, psychological and genetic factors related to maternal and child health in the city of Bradford in the United Kingdom. Of the 317 local authority districts in England, Bradford is the fifth most income deprived, and >33% of its neighbourhoods are among the top 10% most deprived neighbourhoods in England (City of Bradford Metropolitan District Council, 2019). Individuals in BiB thus face barriers to healthy choices including monetary costs, time costs and accessibility, which place them at an elevated risk for disease and poor nutritional status (Darmon & Drewnowski, 2008). As such, all mothers booked for delivery at Bradford's only maternity unit (Bradford Royal Infirmary) between March 2007 and November 2010 were eligible for BiB. BiB recruited 12,453 women (13,776 pregnancies) between 2007 and 2010. Eighty percent of mothers were recruited during the 26‐ to 28‐week visit, while the remainder were recruited during other maternity visits.

All mothers recruited into BiB between August 2008 and March 2009 who had completed the baseline questionnaire were approached to take part in frequent follow‐up assessments through BiB1000. Of the 1916 women recruited to BiB during this time frame, 1735 agreed to take part in the study. Mothers of twin births (*n* = 28) were excluded from this analysis due to differences in growth patterning observed in this cohort (Fairley et al., 2013). Overall follow‐up rates were at least 70% for each visit, with 92% of individuals completing at least one follow‐up assessment over the 36‐month study.

### 2.2 Study measures

Study visits contributing data for analyses were conducted when children were 18 and 36 months of age. Most mothers (61%) preferred to have visits conducted in their homes, with the remainder reporting to research clinics at Bradford Royal Infirmary or local children's centres for some or all visits. All data were collected by interviewers in the participant's preferred language (English, Urdu or Mirpuri) unless otherwise indicated.

### 2.3 HFA of snack foods and SSBs

#### 2.3.1 Measurement

HFA of snack foods and SSBs was assessed using 10 of 39 items from the self‐administered, semi‐quantitative Home Food Availability Inventory Checklist (HFAI‐C) at the 18‐ and 36‐month visits (Table S1) (BiB1000, 2018). Each participant reported whether selected food/beverage items had been present during the past 7 days, and if so, the maximum quantity that had been available (predefined, item‐specific quantities labelled ‘small’, ‘medium’ or ‘large’). Ranges for small, medium and large quantities were provided based on the distribution of sizes of foods/packages that were available from a Universal Product Code (UPC) scanning study (Stevens, Bryant, Wang, Borja, & Bentley, 2011) and on the usual packaging available for purchase in the United Kingdom. The HFAI‐C was developed and validated in a subsample of BiB1000 (Bryant, LeCroy, Sahota, Cai, & Stevens, 2016). Findings indicated that the HFAI‐C has fair to moderate validity (Landis & Koch, 1977) for assessing absence/presence (kappa: 0.39–0.41) and quantity (weighted kappa: 0.25–0.26) of snack foods and beverages (Bryant, LeCroy, Sahota, Cai, & Stevens, 2016).

#### 2.3.2 Variable derivation

HFAI‐C data were used to create two variables each for the snack food and SSB categories: (1) variety and (2) total quantity available in the home in the past week. To determine variety scores, each HFAI‐C item was assigned a score of 0 for absence and 1 for presence, and items were summed within HFAI‐C categories. Thus, variety scores could range from 0 to 7 for snacks and 0 to 3 for SSBs. For quantity, average weight/volume for each item's designated size (e.g. one cup and one medium‐sized can) was calculated by averaging weights/volumes of the specified size of common varieties or brands of each item from the USDA food composition database (Agricultural Research Service, 2018). For items sized by ‘handful’, one handful of nuts or sweets and two handfuls of crisps were considered equivalent to 28.35 g (1 oz). Quantity was determined at the item level by multiplying the average weight/volume by participants' quantity response (median quantity within the designated small, medium or large quantities). Totals of all items within the given category were summed to create a category‐level variable. As a supplementary analysis, information on kilocalories (kcal) was also obtained from the USDA food composition database. Category‐level variables for kcal were created using the same approach described for weight/volume.

### 2.4 Dietary intake

#### 2.4.1 Measurement

Intake of snack foods and SSBs was assessed using validated food frequency questionnaires (FFQs) from the Southampton Women's Survey (Marriott et al., 2009) at 18 months and the Survey of Sugar Intake among Children in Scotland study (Masson et al., 2012) at 36 months. Different FFQs were administered at 18 and 36 months to address the varying nutrition needs at these ages.

Both FFQs were self‐administered and modified to include additional items/prompts appropriate to the study population based on findings from focus groups and 24‐h dietary recalls in Bradford. The 18‐month FFQ included 98 items in 16 categories assessing frequency (never, <1/week, weekly [recorded number of times/week] and >1/day [recorded number of times/day]) and amount of foods/beverages consumed over the preceding month. During administration of the FFQ, pictures and household utensils were used to aid in food recognition and portion size estimations.

The modified 36‐month FFQ included 140 items within 16 categories, asking about the child's diet over the previous 2–3 months. The response categories were ‘rarely or never’, ‘1–2 per week’, ‘1 per week’, ‘2–3 per week’, ‘4–6 per week’, ‘1 per day’, ‘2–3 per day’, ‘4–6 per day’ and ‘7 or more per day’ and were reported in terms of the designated portion size. Examples of food measures were provided in a photograph to help parents estimate the quantities of their child's food intake in standardized terms (e.g. teaspoon, small slice and medium glass). Both the 18‐ and 36‐month FFQs also included a section for participants to list foods/beverages that were not included.

#### 2.4.2 Variable derivation

The list of foods representing snack food and SSB intake in the 18‐ and 36‐month FFQs was not identical due to the use of age‐appropriate, validated FFQs at each time point. Given ambiguity in what is meant by ‘snack’ in British usage (Chamontin, Pretzer, & Booth, 2003), the lack of a consensus definition of ‘snack foods’ in the literature (Johnson & Anderson, 2010) and the lack of clear recommendations for snack intake (Potter, Vlassopoulos, & Lehmann, 2018), the definition of snack foods used in this study was guided by the FFQ‐defined food categories at 18 and 36 months. Use of an FFQ‐based definition also meant that all participants were presented with the same definition of snack foods when completing the questionnaire.

At 18 months, foods were included if they were part of the ‘cakes, biscuits and snacks’ category. Ice cream was also included given it was assessed as a snack food on the HFAI‐C. The 36‐month FFQ did not include sweets in its snack food category (‘crisps, nuts and savoury snacks’), and thus, all items part of the ‘biscuits and cakes’ or ‘sweets, chocolates and ice creams’ categories were included to ensure similar food groups were measured at 18 and 36 months and that the full range of snack foods measured by the HFAI‐C were captured. SSBs were defined at both time points as any non‐dairy, high‐calorie sweetened beverages (Table S1).

Reported frequencies were recalculated to represent intake per week to match the HFAI‐C's measurement time frame. Two variables were derived to describe snack food and SSB intake separately using the same approach described above for the HFAI‐C: (1) variety and (2) total quantity consumed weekly (g/ml and kcal [supplementary analyses]). Due to a large amount of missing data in reporting of portion size consumed on the 18‐month FFQ, quantity was only derived for the 36‐month FFQ. Scores for both variety and quantity were operationalized as tertiles (separately for snacks and SSBs) due to extreme skewedness in the distributions for snack foods and SSBs (cut‐points for each tertile indicated in relevant tables). An additional fourth category was created for variety of SSBs consumed at 18 months to represent non‐consumers of SSBs. There were too few children with no dietary intake of snack foods at 18 months or dietary intake of snack foods or SSBs at 36 months to allow for examination of non‐consumers for these category/age combinations.

### 2.5 Child anthropometrics

Ten study staff trained by expert community researchers measured length/height and weight for all children in BiB1000 (Bryant, Santorelli, et al., 2015). Measurements were taken with the child's clothes removed. All coefficients of reliability indicated good quality control (*r* = 0.96–1.00) (Johnson et al., 2009). Weight was measured using SECA baby scales. Length was measured using the Harlow Health Care neonatometer at 18 months of age, and height was measured using the SECA Leicester height measure at 36 months of age. BMI was calculated as weight (kg) divided by length or height squared (m^2^).

BMI was selected as the primary outcome due to recent literature recommending use of BMI given that BMI *z*‐scores are poor indicators of adiposity in children with obesity (Freedman et al., 2017; Freedman & Berenson, 2017; Kelly & Daniels, 2017). As a sensitivity analysis, weight‐for‐length *z*‐scores at 18 months and BMI *z*‐scores at 36 months were examined. *Z*‐scores were calculated using the World Health Organization's SAS *igrowup* programme (WHO Multicentre Growth Reference Study Group, 2006). Weight‐for‐length *z*‐score was used for 18‐month‐olds given uncertainty in interpreting length‐based BMI *z*‐scores in infancy (Division of Nutrition, Physical Activity, and Obesity, 2015).

### 2.6 Covariates

#### 2.6.1 Ethnicity and immigrant generation

Mother and child's ethnicity were self‐assigned by the mother at baseline using the same classifications as the 2001 Census. Ethnicity was categorized as White, Pakistani or Other, with those of Other ethnicity (*n* = 247) being excluded from the analysis due to heterogeneity of ethnicities within this group and small sample sizes of each ethnicity. Children were classified as either non‐immigrants (all parents and grandparents born in the United Kingdom or Crown dependencies [England, Northern Ireland, Scotland, Wales, Channel Islands or Isle of Man]), second‐generation Pakistani immigrants (at least one parent born in Pakistan) or third‐generation Pakistani immigrants (at least one grandparent born in Pakistan). All other individuals with non‐missing country of origin data were placed in an ‘other immigrants’ group.

#### 2.6.2 Socio‐demographics

Data on maternal age, highest household education, maternal employment status, total number of persons in the household in defined age groups (<2, 2–15, 16–64 and >65 years) and child's sex were collected at baseline using validated items.

### 2.7 Analytic sample

Between 18 (*n* = 1092) and 36 (*n* = 1038) months, 143 Pakistani and White individuals were lost to follow‐up, and 89 who missed the 18‐month visit attended the 36‐month visit. Individuals were excluded at each time point for missing socio‐demographic data (*n* = 18 or 14 at the 18‐ and 36‐month visits, respectively), immigration information (*n* = 10 or 9) and HFA data at the designated time point (*n* = 32 or 29). Individuals missing dietary data or BMI were further excluded from those analyses, as shown in Tables 3, 4, 5. At 18 months, individuals who were excluded were more likely to have a high quantity of snack foods available in the home (3691.5 vs. 2614.4 g). At 36 months, individuals who were excluded were more likely to be younger (26.4 vs. 27.3 years). There were no other significant differences in socio‐demographic or HFA data between individuals who were excluded versus included at each time point.

### 2.8 Statistical analysis

Ethnic and immigrant generational differences in HFA of snack foods and SSBs were examined using separate multinomial logistic regression models. Multinomial instead of ordinal logistic regression models were selected due to violating the proportional odds assumption (Agresti, 2002; McCullagh, 1980). The outcome, HFA scores, was grouped into categories to aid in interpretability of estimates and to account for skewness in the distribution of residuals. HFA variety scores were classified as low (score of 0–2 for snack foods or 1 for SSBs [reference group]), medium (score of 3–5 for snack foods or 2 for SSBs) or high (score of 6–7 for snack foods or 3 for SSBs). A ‘0’ variety score was also created for SSBs at both 18 and 36 months but not for snack foods, given only 19 individuals at 18 months and 9 at 36 months reported not having any snack foods available in the home. HFA quantity for snacks and SSBs, separately, was divided into tertiles, with cut‐points for each tertile indicated in the relevant tables. Exploratory analyses for differences in HFA scores according to immigrant generation were conducted among the Pakistani subgroup with immigrant generation as the main exposure and additional adjustment for mother's age at time of immigration to the United Kingdom.

Cross‐sectional and prospective associations between availability of snack foods and SSBs in the home and dietary intake of snack foods and SSBs (low intake as reference category) were examined using multinomial logistic regression models. Separate models were run for snack foods and SSBs, and two models were examined for each food/beverage category: (1) variety and (2) quantity.

For cross‐sectional analyses, each model used the corresponding HFAI‐C category, measured at the same time as diet, as the main exposure variable. For prospective analysis, each model used the corresponding HFAI‐C category measured at 18 months. Multivariable linear regression models examined cross‐sectional and prospective associations between availability of snack foods and SSBs in the home and BMI. Models were run using weight‐for‐length *z*‐scores and BMI *z*‐scores in sensitivity analyses. Associations with dietary intake and BMI were examined using HFA score as a categorical variable (HFAI‐C categories were derived as previously described). Exploratory analyses were conducted using an interaction between the HFA score and ethnicity for all aforementioned models. Supplemental analyses examined the association between diet at 18 and 36 months to determine whether tracking in diet may underlie the associations between 18‐month HFA and 36‐month diet or BMI.

All regression models were adjusted for the following covariates: child's sex, child's age at time of HFA data collection, mother's age at baseline, mother's education, mother's employment, child's ethnicity and number of individuals in household. These covariates were selected a priori based on previous studies of HFA and diet in children between 18 and 36 months of age (Bryant et al., 2011; Collins, Lacy, Campbell, & McNaughton, 2016; Fernando, Campbell, McNaughton, Zheng, & Lacy, 2018). Models examining prospective associations were additionally adjusted for the child's age at the time of outcome assessment. All analyses were conducted using SAS software, Version 9.4.

### 2.9 Ethical considerations

BiB and BiB1000 were conducted according to the guidelines of the Declaration of Helsinki, and all procedures involving human subjects/patients were approved by the Bradford Research Ethics Committee (07/H1302/112). Written or verbal (for mothers unable to read and/or speak English) informed consent was obtained from all participants. Verbal consent was witnessed and formally recorded.

## 3 RESULTS

An overview of the analytic sample at 18 and 36 months is provided in Table 1. Most households were of low socio‐economic status (~24% of mothers had <5 GCSE equivalent, and ~57% were previously or never employed), and the majority of children were second‐generation Pakistani immigrants (~50%). Pakistani households tended to have a lower socio‐economic status compared with White households (27.3% vs. 20.0% with <5 GSCE equivalent; 22.3% vs. 67.8% currently employed) and more individuals between the ages of 2–15 and 16–64 years living in their households (1.5 vs. 0.7 and 3.3 vs. 2.1, respectively; data not shown in Table 1).

**TABLE 1:** Characteristics of 18‐ and 36‐month‐old children and their families from the Born in Bradford 1000 (BiB1000) study with complete socio‐demographic and home food availability (HFA) data

	18 months (*n* = 1032)	36 months (*n* = 986)
*Socio‐demographics*		
Child's sex, *n* (%)		
Male	505 (48.9)	477 (48.4)
Female	527 (51.1)	509 (51.6)
Child's age (months), mean (SD)	18.7 (1.0)	37.0 (0.9)
Mother's baseline age (years), mean (SD)	27.1 (5.6)	27.3 (5.6)
Mother's education, *n* (%)^a^		
<5 GCSE equivalent	249 (24.1)	231 (23.4)
5 GCSE equivalent	341 (33.0)	325 (33.0)
A‐level equivalent	141 (13.7)	136 (13.8)
Higher than A level	239 (23.2)	230 (23.3)
Other/unknown	62 (6.0)	64 (6.5)
Mother's baseline employment, *n* (%)		
Currently employed	437 (42.3)	428 (43.4)
Previously employed	268 (26.0)	263 (26.7)
Never employed	327 (31.7)	295 (29.9)
Mother's ethnicity, *n* (%)		
White	454 (44.0)	430 (43.6)
Pakistani	578 (56.0)	556 (56.4)
Child's immigrant generation, *n* (%)		
Non‐immigrant	355 (34.3)	338 (34.3)
2nd‐generation Pakistani immigrant	517 (50.1)	499 (50.6)
3rd‐generation Pakistani immigrant	78 (7.6)	70 (7.1)
Non‐Pakistani immigrant	82 (7.9)	79 (8.0)
# individuals in house by age group (years), mean (SD)		
<2	0.2 (0.5)	0.2 (0.5)
2–15	1.1 (1.3)	1.2 (1.3)
16–64	2.8 (1.7)	2.8 (1.6)
≥65	0.1 (0.4)	0.1 (0.3)
*Home food availability, mean (SD)*		
Variety		
Fruits (0–16)	7.8 (3.3)	8.1 (3.1)
Vegetables (0–12 [18 months] or 13 [36 months])	7.0 (2.6)	7.6 (2.8)
Snack foods (0–7)	4.4 (1.7)	4.5 (1.6)
SSBs (0–3)	1.3 (1.0)	1.3 (1.0)
Artificially sweetened beverages (0–1)	0.4 (0.5)	0.3 (0.5)
Quantity (g or ml/week)		
Fruits	5898.6 (3965.6)	7389.3 (4075.7)
Vegetables	4134.7 (3029.2)	4957.1 (3491.8)
Snack foods	2614.4 (1723.1)	2864.4 (1635.3)
Sugar‐sweetened beverages	1973.1 (2194.9)	1882.3 (2088.9)
Artificially sweetened beverages	614.4 (1066.6)	464.8 (888.0)
Quantity (kcal/week)		
Fruits	3725.2 (2429.5)	4665.1 (2453.6)
Vegetables	1350.1 (1013.4)	1609.6 (1159.3)
Snack foods	10,653.5 (6284.8)	11,443.3 (6199.1)
Sugar‐sweetened beverages	663.3 (709.6)	642.7 (688.3)
Artificially sweetened beverages	3.1 (5.3)	2.3 (4.4)
*Obesogenic intake, mean (SD)*	*n* = 1020^b^	*n* = 971^b^
Variety		
Snack foods (0–7 or 20)	4.2 (1.5)	11.3 (4.1)
Sugar‐sweetened beverages (0–4 or 6)	1.1 (1.0)	3.1 (1.8)
Quantity (g or ml/week)		
Snack foods	‐	904.6 (985.5)
Sugar‐sweetened beverages	‐	2814.1 (3919.0)
Quantity (kcal/week)		
Snack foods	‐	3589.3 (3631.3)
Sugar‐sweetened beverages	‐	1163.9 (1686.5)
*Body measurements, mean (SD)*	*n* = 921^b^	*n* = 816^b^
Body mass index (kg/m^2^)	16.2 (1.5)	16.3 (1.5)
Weight‐for‐length *z*‐score	0.2 (1.0)	‐
Body mass index *z*‐score	‐	0.5 (1.0)

Abbreviation: GCSE, General Certificate of Secondary Education.

^a^ Note that each GCSE exam is a test of knowledge in a particular subject (e.g. mathematics). Passing >5 GCSEs is comparable to a high school diploma in the United States. An A‐level equivalent is comparable to a high school diploma with a transcript that includes advanced courses (i.e. advancement placement or International Baccalaureate courses).

^b^ Denotes a smaller sample size due to missing food frequency questionnaire or body mass index data.

Table 2 shows the adjusted estimates for ethnic differences in HFA of snack foods and SSBs. Results were largely consistent at 18 and 36 months, with White homes having significantly lower odds of having a high versus low variety or quantity of snack foods or SSBs in their home compared with Pakistani homes. In addition, the odds of having no SSBs versus a low variety of SSBs available were 1.72 (95% CI: 1.11, 2.66) and 2.81 (95% CI: 1.76, 4.49) times greater for White compared with Pakistani households at 18 and 36 months, respectively. No differences in HFA of snack foods or SSBs were observed according to immigrant generation (data not shown).

**TABLE 2:** Adjusted odds ratios (95% CIs) for race/ethnic differences in home food availability (medium or high vs. low) of snack foods and sugar‐sweetened beverages (SSBs), by variety and quantity, among 18‐ and 36‐month‐old children in White compared with Pakistani households^a^

	Home food availability cut‐points	
Snack foods		
*Variety*		
*18 months (n = 1032)*	*Medium (3–5 items)*	*High (6–7 items)*
Pakistani	Ref.	Ref.
White	0.71 (0.44, 1.16)	0.44 (0.25, 0.75)**
*36 months (n = 986)*	*Medium (3–5 items)*	*High (6–7 items)*
Pakistani	Ref.	Ref.
White	0.76 (0.45, 1.29)	0.37 (0.21, 0.66)**
*Quantity*		
*18 months (n = 1032)*	*Medium (>1772 to ≤2896 g)*	*High (>2896 g)*
Pakistani	Ref.	Ref.
White	0.60 (0.41, 0.89)*	0.43 (0.29, 0.65)**
*36 months (n = 986)*	*Medium (>2001 to ≤3039 g)*	*High (>3039 g)*
Pakistani	Ref.	Ref.
White	0.70 (0.47, 1.04)	0.45 (0.30, 0.69)**
SSBs		
*Variety*		
*18 months (n = 1032)*	*Medium (2 items)*	*High (3 items)*
Pakistani	Ref.	Ref.
White	0.70 (0.46, 1.07)	0.27 (0.15, 0.49)**
*36 months (n = 986)*	*Medium (2 items)*	*High (3 items)*
Pakistani	Ref.	Ref.
White	0.41 (0.26, 0.63)**	0.22 (0.12, 0.41)**
*Quantity*		
*18 months (n = 1032)*	*Medium (>654 to ≤1800 ml)*	*High (>1800 ml)*
Pakistani	Ref.	Ref.
White	0.58 (0.39, 0.85)**	0.35 (0.23, 0.53)**
*36 months (n = 986)*	*Medium (>654 to ≤1800 ml)*	*High (>1800 ml)*
Pakistani	Ref.	Ref.
White	0.34 (0.22, 0.51)**	0.22 (0.14, 0.33)**

*Note*: Separate models were run for snack foods and SSBs, and two models were examined for each food/beverage category: one for variety and another for total quantity. Variety of SSBs in the home had an additional category of ‘none’ at both 18 and 36 months. To improve clarity in interpreting the above table, the odds ratios for having no SSBs versus a low variety of SSBs in the home are only listed here: 1.72 (95% CI: 1.11, 2.66) for White versus Pakistani households at 18 months and 2.81 (95% CI: 1.76, 4.49) for White versus Pakistani households at 36 months.

^a^ All multinomial logistic regression models adjusted for the following covariates: child's sex, child's age, mother's baseline age, mother's education, mother's baseline employment status, mother's ethnicity and household size.

* *p* < 0.05.

** *p <* 0.01.

Tables 3 and 4 show the adjusted estimates for the association between availability of snack foods and SSBs in the home and dietary intake. Both tables show that greater HFA (variety and quantity) of snack foods and SSBs was associated with greater intake (variety and quantity) cross‐sectionally at 18 and 36 months. Associations for availability of snack foods and SSBs (variety and quantity) in the home at 18 months with dietary intake at 36 months were also generally statistically significant in the expected direction. The odds of having a high versus low intake of a variety of snack foods or SSBs at 36 months was 1.27 (95% CI: 1.14, 1.42) or 1.47 (95% CI: 1.20, 1.79) times greater for every additional type of snack food or SSB made available in the home at 18 months, respectively. Similarly, having a large compared with small quantity of snack foods or SSBs in the home at 18 months was associated with a 2.06 (95% CI: 1.30, 3.25) or 2.71 (95% CI: 1.73, 4.26) times greater odds of consuming a high versus low quantity of snack foods or SSBs, respectively, at 36 months.

**TABLE 3:** Cross‐sectional and prospective associations between home food availability (HFA) of snack foods (variety and quantity) and snack food intake (medium or high vs. low) among children at 18 and 36 months of age in Pakistani and White households, Born in Bradford 1000 (BiB1000) study^a^

HFA at designated time (exposure)	Categories of snack food intake by variety or quantity (outcome)	
	Medium	High
Cross‐sectional analyses		
*18 months (n = 1020)* ^b^		
Variety in HFA of snack foods	3–5 FFQ items	6–7 FFQ items
Low (0–2 items)	1.00	1.00
Medium (3–5 items)	2.76 (1.72, 4.44)**	4.55 (2.30, 8.99)**
High (6–7 items)	4.29 (2.32, 7.93)**	13.82 (6.29, 30.40)**
*36 months (n = 971)* ^c^		
Variety in HFA of snack foods	>9 to ≤13 FFQ items	>13 FFQ items
Low (0–2 items)	1.00	1.00
Medium (3–5 items)	3.20 (1.93, 5.31)**	2.43 (1.37, 4.33)*
High (6–7 items)	8.68 (4.82, 15.64)**	15.32 (8.11, 28.94)**
Quantity for HFA of snack foods	>446 to ≤876 g, FFQ	>876 g, FFQ
Low (≤2001 g)	1.00	1.00
Medium (>2001 to ≤3939 g)	2.19 (1.52, 3.16)**	2.29 (1.49, 3.52)**
High (>3039 g)	3.40 (2.20, 5.26)**	7.06 (4.46, 11.17)**
Prospective analyses		
*18 months (n = 882)* ^d^		
Variety in HFA of snack foods	>9 to ≤13 FFQ items	>13 FFQ items
Low (0–2 items)	1.00	1.00
Medium (3–5 items)	1.26 (0.80, 2.00)	1.93 (1.12, 3.35)*
High (6–7 items)	1.93 (1.14, 3.27)*	3.16 (1.72, 5.79)**
Quantity for HFA of snack foods	>442 to ≤876 g, FFQ	>876 g, FFQ
Low (≤1772 g)	1.00	1.00
Medium (>1772 to ≤2898 g)	1.42 (0.96, 2.09)	1.86 (1.19, 2.89)**
High (>2898 g)	1.17 (0.76, 1.78)	2.06 (1.30, 3.25)**

Abbreviation: FFQ, food frequency questionnaire.

^a^ All multinomial logistic regression models adjusted for the following covariates: child's sex, child's age, mother's baseline age, mother's education, mother's baseline employment status, mother's ethnicity and household size.

^b^ 18‐month cross‐sectional analyses (this section of rows) used HFA and diet data from 18 months.

^c^ 36‐month cross‐sectional analyses (this section of rows) used HFA and diet data from 36 months.

^d^ Prospective analyses (this section of rows) used HFA data from 18 months and diet data from 36 months.

* *p* < 0.05.

** *p* < 0.01.

**TABLE 4:** Cross‐sectional and prospective associations between home food availability (HFA) of sugar‐sweetened beverages (SSBs; variety and quantity) and SSB intake (medium or high vs. low) among children 18 and 36 months of age in Pakistani and White households, Born in Bradford 1000 (BiB1000) study^a^

HFA at designated time (exposure)	Categories of SSB intake by variety or quantity (outcome)	
	Medium	High
Cross‐sectional analyses		
*18 months (n = 1020)* ^b^		
Variety in HFA of SSBs	2 FFQ items*	3–4 FFQ items
No SSBs (0 items)	0.88 (0.56, 1.40)	0.90 (0.29, 2.80)
Low (1 item)	1.00	1.00
Medium (2 items)	1.12 (0.75, 1.69)	5.65 (2.59, 12.34)**
High (3 items)	1.30 (0.79, 2.14)	5.68 (2.39, 13.51)**
*36 months (n = 971)* ^c^		
Variety in HFA of SSBs	>2 to ≤4 FFQ items	>4 FFQ items
No SSBs (0 items)	0.44 (0.30, 0.66)**	0.25 (0.14, 0.45)**
Low (1 item)	1.00	1.00
Medium (2 items)	1.67 (1.11, 2.51)*	3.08 (1.94, 4.88)**
High (3 items)	1.81 (1.03, 3.16)*	4.71 (2.61, 8.49)**
Quantity for HFA of SSBs	>889 to ≤2649 ml, FFQ	>2649 ml, FFQ
Low (≤654 ml)	1.00	1.00
Medium (>654 to ≤1800 ml)	2.16 (1.46, 3.20)**	1.88 (1.24, 2.84)**
High (>1800 ml)	2.53 (1.65, 3.90)**	4.24 (2.76, 6.50)**
Prospective analyses		
*18 months (prospective) (n = 882)* ^d^		
Variety in HFA of SSBs	>2 to ≤4 FFQ items	>4 FFQ items
No SSBs (0 items)	0.69 (0.46, 1.04)	0.66 (0.40, 1.09)
Low (1 item)	1.00	1.00
Medium (2 items)	0.90 (0.60, 1.36)	1.16 (0.73, 1.85)
High (3 items)	1.79 (1.01, 3.16)*	2.59 (1.40, 4.78)**
Quantity for HFA of SSBs	>887 to ≤2569 ml, FFQ	>2569 ml, FFQ
Low (≤654 ml)	1.00	1.00
Medium (>654 to ≤1800 ml)	1.29 (0.86, 1.94)	1.48 (0.98, 2.25)
High (>1800 ml)	1.94 (1.24, 3.04)*	2.71 (1.73, 4.26)**

*Note*: Variety of SSBs consumed at 18 months had an additional category of ‘none’. To improve clarity in interpreting the above table, the significant results of cross‐sectional analyses examining the odds ratios for having no consumption versus a low consumption of a variety of SSBs at 18 months are only listed here: 1.97 (95% CI: 1.32, 2.94) for having no types versus a low variety of SSBs in the home at 18 months and 0.51 (95% CI: 0.27, 0.96) for having a high versus low variety of SSBs in the home at 18 months.

Abbreviation: FFQ, food frequency questionnaire.

^a^ All multinomial logistic regression models adjusted for the following covariates: child's sex, child's age, mother's baseline age, mother's education, mother's baseline employment status, mother's ethnicity and household size.

^b^ 18‐month cross‐sectional analyses (this section of rows) used HFA and diet data from 18 months.

^c^ 36‐month cross‐sectional analyses (this section of rows) used HFA and diet data from 36 months.

^d^ Prospective analyses (this section of rows) used HFA data from 18 months and diet data from 36 months.

* *p* < 0.05.

** *p* < 0.01.

Adjusted associations between HFA of snack foods and SSBs and BMI are shown in Table 5 and indicate primarily null associations. The associations did not significantly change when weight‐for‐length *z*‐score (18 months) or BMI *z*‐score (36 months) was used as the outcome (Table S2). Associations were unchanged when kcal was used in place of g/ml (Tables S3–S6). Positive associations between diet at 18 and 36 months were observed in Table S7. In exploratory analyses of effect modification, associations of HFA with dietary intake were not consistently modified by ethnicity, and no effect modification was observed by ethnicity for associations of HFA with BMI.

**TABLE 5:** Cross‐sectional and prospective associations between home food availability (HFA) of snack foods or sugar‐sweetened beverages (SSBs; variety and quantity) and body mass index (BMI) among children at 18 or 36 months of age in Pakistani and White households, Born in Bradford 1000 (BiB1000) study^a^

	Time of BMI measurement (outcome)					
	Cross‐sectional: 18 months (*n* = 921)^b^		Cross‐sectional: 36 months (*n =* 816)^c^		Prospective: 36 months (*n* = 743)^d^	
	HFA cut‐points	*β* (95% CIs)	HFA cut‐points	*β* (95% CIs)	HFA cut‐points	*β* (95% CIs)
Snack foods						
*Variety*						
Low	0–2 items	0.00	0–2 items	0.00	0–2 items	0.00
Medium	3–5 items	−0.14 (−0.42, 0.15)	3–5 items	0.31 (−0.01, 0.62)	3–5 items	−0.28 (−0.60, 0.03)
High	6–7 items	−0.21 (−0.53, 0.10)	6–7 items	0.38 (0.03, 0.72)*	6–7 items	−0.28 (−0.64, 0.07)
*Quantity*						
Low	≤1772 g	0.00	≤2001 g	0.00	≤1772 g	0.00
Medium	>1772 to ≤2898 g	−0.05 (−0.28, 0.18)	>2001 to ≤3039 g	0.06 (−0.19, 0.31)	>1772 to ≤2881 g	−0.08 (−0.34, 0.19)
High	>2898 g	−0.14 (−0.38, 0.11)	>3039 g	0.27 (0.01, 0.53)*	>2881 g	−0.25 (−0.53, 0.02)
SSBs						
*Variety*						
No SSBs	0 items	−0.13 (−0.38, 0.12)	0 items	−0.03 (−0.30, 0.24)	0 items	0.08 (−0.20, 0.36)
Low	1 item	0.00	1 item	0.00	1 item	0.00
Medium	2 items	−0.27 (−0.52, −0.03)*	2 items	−0.02 (−0.28, 0.24)	2 item	−0.20 (−0.47, 0.08)
High	3 items	−0.17 (−0.48, 0.14)	3 items	0.37 (0.03, 0.70)*	3 items	0.01 (−0.35, 0.38)
*Quantity*						
Low	≤654 ml	0.00	≤654 ml	0.00	≤654 ml	0.00
Medium	>654 to ≤1800 ml	−0.21 (−0.45, 0.02)	>654 to ≤1747 ml	0.07 (−0.18, 0.32)	>654 to ≤1747 ml	−0.31 (−0.58, −0.04)*
High	>1800 ml	−0.15 (−0.40, 0.09)	>1747 ml	0.18 (−0.08, 0.43)	>1747 ml	−0.11 (−0.37, 0.16)

^a^ All multivariable linear regression models adjusted for the following covariates: child's sex, child's age, mother's baseline age, mother's education, mother's baseline employment status, mother's ethnicity and household size.

^b^ 18‐month cross‐sectional analyses (this column) used HFA and BMI data from 18 months.

^c^ 36‐month cross‐sectional analyses (this column) used HFA and BMI data from 36 months.

^d^ Prospective analyses (this column) used HFA data from 18 months and BMI data from 36 months.

* *p* < 0.05.

** *p* < 0.01.

## 4 DISCUSSION

This is the largest study to examine the HFA of snack foods and SSBs in Pakistani and White households and the first to assess the association of HFA of snack foods and SSBs during the first 1000 days with obesogenic dietary intake and BMI in early childhood in any ethnicity. In a sample of Pakistani and White families living in Bradford, UK, Pakistani homes had a greater variety and quantity of snack foods and SSBs available in the home when toddlers were 18 and 36 months of age. Irrespective of ethnicity, greater availability of snack foods and SSBs in the home during the first 1000 days was associated with increased intake of snack foods and SSBs at 36 months of age, but not with BMI.

The observed ethnic differences in HFA of SSBs are consistent with previous research conducted in a small subsample (*n* = 97) of BiB1000 at 18 months (Bryant, Sahota, et al., 2015), with the current study additionally finding ethnic differences in HFA of snack foods. Previous studies have shown that HFA varies according to race/ethnicity during toddlerhood (Fuemmeler et al., 2015; Larson, Eisenberg, Berge, Arcan, & Neumark‐Sztainer, 2015), but this study is the first to report differences in HFA of Pakistani and White households at 36 months of age. Contrary to the hypothesis, there were no differences in HFA of snack foods and SSBs according to immigrant generation.

Findings for ethnic differences in HFA of snack foods and SSBs are also similar to ethnic differences in dietary intake previously observed in BiB1000 at 18 and 36 months (Mahoney, Bryant, Sahota, & Barber, 2018; Sahota et al., 2016). Thus, it is not surprising that the variety and quantity of snack foods and SSBs available in the home were positively associated with dietary intake at 18 and 36 months and in prospective analyses. Previous cross‐sectional examinations of preschool‐aged children have indicated a positive association between HFA of snack foods and SSBs and dietary intake (McGowan, Croker, Wardle, & Cooke, 2012; Spurrier et al., 2008). However, this is the first study, to our knowledge, to examine cross‐sectional associations of the HFA of snack foods and SSBs and diet at 18 months of age. Given that the period between 12 and 24 months of age is when children complete the transition from breastfeeding or formula to table food (Birch & Doub, 2014), this study's findings provide new insight into an important determinant of toddler's dietary intake during this critical dietary transition period.

Only two previous studies, both conducted using data from the Melbourne Infant Feeding, Activity and Nutrition Trial in Australia, have explored the relationship between HFA during the first 1000 days and later dietary intake, but neither examined HFA quantities. They found that having snack foods in the home more frequently at 18 months of age was associated with greater overall dietary energy density at 3.5 years of age (Fernando, Campbell, McNaughton, Zheng, & Lacy, 2018) and that increased frequency of having fruits available in the home was linked to a lower overall dietary energy density (Fernando, Campbell, McNaughton, Zheng, & Lacy, 2018) and higher overall diet quality (Collins, Lacy, Campbell, & McNaughton, 2016) at follow‐up. Though the measures of HFA and diet used in the present study differed, together these findings support a significant prospective association between HFA at 18 months and diet during toddlerhood.

Future research should examine potential mediators of this association, including 18‐month dietary intake. Early‐life HFA may affect 36‐month dietary intake by influencing dietary preferences at 18 months that track through childhood. However, such research needs to consider that children's diets are susceptible to changing over time as their energy needs and demand for specific nutrients change (Public Health England, 2016). This is particularly true for the age range under study, given that 2‐ to 5‐year‐olds in the United Kingdom are advised to begin following adult dietary recommendations, which are distinct from those for 12‐ to 24‐month‐olds (National Health Service, 2019; Public Health England, 2019). Additionally, serving sizes for 1‐ to 3‐year‐olds are approximately a quarter of those for adults (American Academy of Pediatrics: Committee on Nutrition, 2016), with one serving equalling one half to two biscuits (6–23 g) or 100–120 ml of SSBs (Infant and Toddler Forum, 2014). As such, future prospective studies need to account for the variations in both the types and quantities of foods children consume across this age range.

Future prospective and intervention studies should also consider the potential moderating role of parents' behaviours in the association between early‐life HFA and youth's later dietary intake/obesity risk (Savage, Fisher, & Birch, 2007). Parents' purchasing behaviours and their knowledge of healthy food choices determine what foods/beverages parents make available and accessible to their children (Campbell et al., 2013; Savage, Fisher, & Birch, 2007). Further, parenting styles and food parenting practices affect both HFA and when/if snacks and SSBs are offered to children (Gable & Lutz, 2000; Patrick, Nicklas, Hughes, & Morales, 2005; Savage, Fisher, & Birch, 2007). Examining how these parental behaviours relate to the HFA of snacks and SSBs and youth's dietary intake may provide important insight into future intervention targets.

Despite the positive association between HFA of snack foods and SSBs and dietary intake, associations with BMI were primarily null. HFAI‐C snack food and SSB items may not be major contributors to dietary intake in the BiB1000 sample, and 1‐week HFA may not capture usual HFA (Sharkey, Dean, St John, & Huber, 2010; Sisk, Sharkey, McIntosh, & Anding, 2010). HFA is also only one of many risk factors shown to influence children's risk for obesity. Other risk factors including maternal behaviours during pregnancy, breastfeeding, food parenting practices, foods outside the home, sleep and physical activity may be more strongly associated with risk for obesity in this sample and should be considered in future research (Dev, McBride, Fiese, Jones, & Cho, 2013; Woo Baidal et al., 2016).

### 4.1 Strengths and limitations

This study was conducted in a large bi‐ethnic sample of low‐income Pakistani and White households. Socio‐demographic and dietary data were collected using culturally appropriate, validated questionnaires, and trained study personnel measured all anthropometrics. Use of the HFAI‐C as opposed to other HFA assessment tools allowed us to examine previously under‐researched aspects of the home food environment, specifically variety and quantity of foods/beverages available in the home.

However, results from this study may not be applicable to other South Asian ethnicities. Both FFQs included more than 100 questions, which placed a high burden on participants that could have led to inaccurate dietary recall (Willett, 2012). It is also possible that the weights/volumes of some FFQ items were not correctly specified due to assumptions regarding what constitutes a ‘small’ portion size and use of a US‐based tool for estimating weights of foods from the United Kingdom. Similarly, supplementary analyses involving kcal relied on multiple assumptions, including that (1) all brands have the same nutritional value and (2) the nutritional value of commercially produced foods is the same in the United States as in the United Kingdom. Additionally, snack foods of varying nutritional composition were collapsed into a single category for analyses, which prevented conclusions regarding how HFA is associated with toddler's macronutrient intake.

## 5 CONCLUSIONS

In this study of Pakistani and White toddlers, Pakistani households had more obesogenic foods and beverages available in their homes during toddlerhood compared with White households. Findings suggest that reducing the HFA of snack foods and SSBs in early toddlerhood may help promote the development of a more healthful diet among Pakistani and White children, but future studies are needed to determine how HFA may interact with other early childhood obesity risk factors to affect adiposity.

## CONFLICTS OF INTEREST

The authors declare that they have no conflicts of interest.

## CONTRIBUTIONS

MNL was the primary author and analysed and interpreted all data. JS is the senior author and oversaw and contributed to all aspects of the manuscript completion and was the primary contributor to manuscript revisions. MB aided with data collection and, along with SSA, AMSR, DSW and JC, provided revisions to previous drafts of this manuscript. JC helped design the analytic plan. All authors contributed to the research question design and read and approved the final manuscript.

## Supporting information


**Table S1.** Food and beverage items used to define “snack foods” and “SSBs” in the Home Food Availability Inventory Checklist (HFAI‐C) and food frequency questionnaires (FFQs) implemented in the Born in Bradford 1000 (BiB1000) study^†^
**Table S2.** Cross‐sectional and prospective associations between home food availability (HFA) of snack foods or sugar‐sweetened beverages (SSBs; variety and quantity) with child's weight‐for‐length z‐score at 18 months of age or body mass index (BMI) z‐score at 36 months of age in Pakistani and White households, Born in Bradford 1000 (BiB1000) study^†^
**Table S3.** Adjusted odds ratios (95% CIs) for race/ethnic differences in home food availability (medium or high vs. low) of snack foods and sugar‐sweetened beverages (SSBs) among 18‐ and 36‐month old children in White compared to Pakistani households^†^
**Table S4.** Cross‐sectional and prospective associations between home food availability (HFA) of snack foods (quantity) and snack food intake (medium or high vs. low) among children at 18 and 36 months of age in Pakistani and White households, Born in Bradford 1000 (BiB1000) study^†^
**Table S5.** Cross‐sectional and prospective associations between home food availability (HFA) of sugar‐sweetened beverages (SSBs; quantity) and SSB intake (medium or high vs. low) among children 18 and 36 months of age in Pakistani and White households, Born in Bradford 1000 (BiB1000) study^†^
**Table S6.** Cross‐sectional and prospective associations between home food availability (HFA) of snack foods or sugar‐sweetened beverages (SSBs; variety and quantity) and body mass index (BMI) among children at 18 and 36 months of age in Pakistani and White households, Born in Bradford 1000 (BiB1000) study^†^
**Table S7.** Prospective association between variety of snack foods and sugar‐sweetened beverages (SSBs) consumed at 18 months and their respective intake (medium or high vs. low) at 36 months among children in Pakistani and White households, Born in Bradford 1000 (BiB1000) study^†^
