# Supplementary material for: Obesogenic home food availability, diet, and BMI in Pakistani and White toddlers
Source: Matern Child Nutr. 2021 Jan 19;17(3):e13138. doi: 10.1111/mcn.13138 (PMC8189220; doi:10.1111/mcn.13138)
Supplement: Supplementary file 1 — Table S1. Food and beverage items used to define “snack foods” and “SSBs” in the Home Food Availability Inventory Checklist (HFAI‐C) and food frequency questionnaires (FFQs) implemented in the Born in Bradford 1000 (BiB1000) study† Table S2. Cross‐sectional and prospective associations between home food availability (HFA) of snack foods or sugar‐sweetened beverages (SSBs; variety and quantity) with child's weight‐for‐length z‐score at 18 months of age or body mass index (BMI) z‐score at 36 months of age in Pakistani and White households, Born in Bradford 1000 (BiB1000) study† Table S3. Adjusted odds ratios (95% CIs) for race/ethnic differences in home food availability (medium or high vs. low) of snack foods and sugar‐sweetened beverages (SSBs) among 18‐ and 36‐month old children in White compared to Pakistani households† Table S4. Cross‐sectional and prospective associations between home food availability (HFA) of snack foods (quantity) and snack food intake (medium or high vs. low) among children at 18 and 36 months of age in Pakistani and White households, Born in Bradford 1000 (BiB1000) study† Table S5. Cross‐sectional and prospective associations between home food availability (HFA) of sugar‐sweetened beverages (SSBs; quantity) and SSB intake (medium or high vs. low) among children 18 and 36 months of age in Pakistani and White households, Born in Bradford 1000 (BiB1000) study† Table S6. Cross‐sectional and prospective associations between home food availability (HFA) of snack foods or sugar‐sweetened beverages (SSBs; variety and quantity) and body mass index (BMI) among children at 18 and 36 months of age in Pakistani and White households, Born in Bradford 1000 (BiB1000) study† Table S7. Prospective association between variety of snack foods and sugar‐sweetened beverages (SSBs) consumed at 18 months and their respective intake (medium or high vs. low) at 36 months among children in Pakistani and White households, Born in Bradford 1000 (BiB1000) study† [file MCN-17-e13138-s001.docx]

**Supplementary Table 1.** Food and beverage items used to define “snack foods” and “SSBs” in the Home Food Availability Inventory Checklist (HFAI-C) and food frequency questionnaires (FFQs) implemented in the Born in Bradford 1000 (BiB1000) study^†^

|  |  | **HFAI-C** | **FFQ items** | |
| --- | --- | --- | --- | --- |
|  |  |  | **18-month** | **36-month** |
| **Snack foods** |  | 1. Crisps and tortilla chips | 1. Crisps and savory snacks | 1. Regular crisps |
|  |  |  |  | 2. Reduced fat crisps |
|  |  |  |  | 3. Other savory snacks |
|  |  |  |  | 4. Savory biscuits, crackers, or breadsticks |
|  |  | 2. Salted nuts |  | 5. Peanuts and other nuts |
|  |  | 3. Cakes and muffins | 2. Cakes, buns, and pastries | 6. Plain cakes |
|  |  |  |  | 7. Cakes with icing |
|  |  |  |  | 8. Cream cakes or gateaux |
|  |  |  |  | 9. Fruit cake or malt loaf |
|  |  |  |  | 10. Doughnuts, muffins, or pastries |
|  |  |  |  | 11. Cereal bars or flapjacks |
|  |  |  |  | 12. Scones or pancakes |
|  |  | 4. Biscuits | 3. Chocolate and digestive biscuits | 13. Chocolate biscuits or cookies |
|  |  |  | 4. Other biscuits | 14. Plain biscuits |
|  |  |  |  | 15. Fancy biscuits |
|  |  | 5. Chocolate | 5. Chocolate | 16. Chocolate bars |
|  |  | 6. Sweets | 6. Sweets | 17. Boiled, chewy, or chocolate sweets |
|  |  | 7. Ice cream | 7. Ice cream | 18. Wrapper ice creams |
|  |  |  |  | 19. Other ice cream |
|  |  |  |  | 20. Iced lollies |
|  |  |  |  |  |
| **SSBs** |  | 1. Fruit drinks | 1. Fruit drinks | 1. High juice fruit drinks |
|  |  |  | 2. Ribena | 2. Regular fruit juice drinks |
|  |  |  | 3. Squash | 3. Regular blackcurrant diluting juice |
|  |  |  |  | 4. Regular orange, lemon, or other diluting juice |
|  |  |  |  | 5. Other fruit-flavored drinks |
|  |  | 2. Fizzy drinks | 4. Regular fizzy drinks | 6. Regular fizzy drinks |
|  |  | 3. Sports drinks |  |  |

American equivalents for British terminology: crisps=chips; gateaux=cake with cream or fruit; malt loaf=sweet leavened bread; flapjacks=baked oat bars; biscuits=cookies; sweets=candies; iced lollies=popsicles; Ribena=fruit-flavored soft drink in the United Kingdom; squash or diluting juice=fruit-flavored concentrate used to make beverages

^†^*SSBs* sugar-sweetened beverages

**Supplementary Table 2.** Cross-sectional and prospective associations between home food availability (HFA) of snack foods or sugar-sweetened beverages (SSBs; variety and quantity) with child’s weight-for-length z-score at 18 months of age or body mass index (BMI) z-score at 36 months of age in Pakistani and White households, Born in Bradford 1000 (BiB1000) study^†^

|  | **Time of body composition measurement (Outcome)** | | | | | | | |
| --- | --- | --- | --- | --- | --- | --- | --- | --- |
|  | **Cross-sectional:**  **18 months (*n*=921)**  ***Weight-for-length z-score***^‡^ | |  | **Cross-sectional:**  **36 months (*n=*816)**  ***BMI z-score***^§^ | |  | **Prospective:**  **36 months (*n*=743)**  ***BMI z-score***^¶^ | |
|  | HFA  cut-points | β (95% CIs) |  | HFA  cut-points | β (95% CIs) |  | HFA  cut-points | β (95% CIs) |
| **Snack foods** |  |  |  |  |  |  |  |  |
| Variety |  |  |  |  |  |  |  |  |
| Low | 0-2 items | 0.00 |  | 0-2 items | 0.00 |  | 0-2 items | 0.00 |
| Medium | 3-5 items | -0.10 (-0.30, 0.11) |  | 3-5 items | 0.23 (0.01, 0.45)* |  | 3-5 items | -0.20 (-0.43, 0.02) |
| High | 6-7 items | -0.16 (-0.39, 0.06) |  | 6-7 items | 0.28 (0.04, 0.52)* |  | 6-7 items | -0.19 (-0.44, 0.06) |
| Quantity |  |  |  |  |  |  |  |  |
| Low | ≤1772g | 0.00 |  | ≤2001g | 0.00 |  | ≤1772g | 0.00 |
| Medium | >1772 to  ≤2898g | -0.04 (-0.21, 0.13) |  | >2001 to  ≤3039g | 0.05 (-0.13, 0.22) |  | >1772 to  ≤2881g | -0.07 (-0.25, 0.12) |
| High | >2898g | -0.11 (-0.29, 0.07) |  | >3039g | 0.20 (0.02, 0.39)* |  | >2881g | -0.18 (-0.37, 0.02) |
| **SSBs** |  |  |  |  |  |  |  |  |
| Variety |  |  |  |  |  |  |  |  |
| No SSBs | 0 items | -0.10 (-0.28, 0.08) |  | 0 items | -0.03 (-0.22, 0.16) |  | 0 items | 0.04 (-0.16, 0.24) |
| Low | 1 item | 0.00 |  | 1 item | 0.00 |  | 1 item | 0.00 |
| Medium | 2 items | -0.19 (-0.36, -0.01)* |  | 2 items | -0.02 (-0.20, 0.17) |  | 2 item | -0.13 (-0.32, 0.07) |
| High | 3 items | -0.13 (-0.35, 0.09) |  | 3 items | 0.26 (0.03, 0.50)* |  | 3 items | 0.001 (-0.25, 0.26) |
| Quantity |  |  |  |  |  |  |  |  |
| Low | ≤654mL | 0.00 |  | ≤654mL | 0.00 |  | ≤654mL | 0.00 |
| Medium | >654 to  ≤1800mL | -0.13 (-0.30, 0.04) |  | >654 to  ≤1747mL | 0.05 (-0.13, 0.23) |  | >654 to  ≤1747mL | -0.20 (-0.39, -0.01)* |
| High | >1800mL | -0.11 (-0.28, 0.06) |  | >1747mL | 0.13 (-0.05, 0.31) |  | >1747mL | -0.07 (-0.25, 0.12) |

*p<0.05, **p<0.01

^†^All multivariable linear regression models adjusted for the following covariates: child’s sex, child’s age, mother’s baseline age, mother’s education, mother’s baseline employment status, mother’s ethnicity, and household size

^‡^18-month cross-sectional analyses (this column) used HFA and weight-for-length z-score data from 18 months

^§^36-month cross-sectional analyses (this column) used HFA and BMI z-score data from 36 months

^¶^Prospective analyses (this column) used HFA data from 18 months and BMI z-score data from 36 months

**Supplementary Table 3.** Adjusted odds ratios (95% CIs) for race/ethnic differences in home food availability (medium or high vs. low) of snack foods and sugar-sweetened beverages (SSBs) among 18- and 36-month old children in White compared to Pakistani households^†^

|  | **Home food availability cut-points** | | | |
| --- | --- | --- | --- | --- |
| **Snack foods** |  | |  | |
| ***Quantity (kcal)*** |  | |  | |
| *18 months (n=1032)* | *Medium (>8610 to ≤11451 kcal)* | | *High (>11451 kcal)* | |
| Pakistani | Ref. | | Ref. | |
| White | 0.67 (0.45, 0.99)* | | 0.42 (0.28, 0.63)** | |
| *36 months (n=986)* | *Medium (>9266 to ≤12204 kcal)* | | *High (>12204 kcal)* | |
| Pakistani | Ref. | | Ref. | |
| White | 0.59 (0.40, 0.88)** | | 0.42 (0.28, 0.64)** | |
| **SSBs** |  | |  | |
| ***Quantity (kcal)*** |  |  | |  |
| *18 months (n=1032)* | *Medium (>230 to ≤660 kcal)* | | *High (>660 kcal)* | |
| Pakistani | Ref. | | Ref. | |
| White | 0.57 (0.39, 0.85)** | | 0.35 (0.23, 0.53)** | |
| *36 months (n=986)* | *Medium (>230 to ≤660 kcal)* | | *High (>660 kcal)* | |
| Pakistani | Ref. | | Ref. | |
| White | 0.34 (0.22, 0.51)** | | 0.22 (0.14, 0.33)** | |

*p<0.05, **p<0.01

Separate models were run for snack foods and SSBs.

^†^All multinomial logistic regression models adjusted for the following covariates: child’s sex, child’s age, mother’s baseline age, mother’s education, mother’s baseline employment status, mother’s ethnicity, and household size

**Supplementary Table 4.** Cross-sectional and prospective associations between home food availability (HFA) of snack foods (quantity) and snack food intake (medium or high vs. low) among children at 18 and 36 months of age in Pakistani and White households, Born in Bradford 1000 (BiB1000) study^†^

| **HFA at designated time (Exposure)** | **Categories of snack food intake quantity (Outcome)** | |
| --- | --- | --- |
|  | Medium | High |
| **Cross sectional analyses** |  |  |
| ***36 months*** *(n=971)*^‡^ |  |  |
| Quantity for HFA of snack foods | >1835 to ≤3532 kcal, FFQ^§^ | >3532 kcal, FFQ |
| Low (≤9267 kcal) | 1.00 | 1.00 |
| Medium (>9267 to ≤12204 kcal) | 2.34 (1.62, 3.39)** | 2.73 (1.79, 4.17)** |
| High (>12204 kcal) | 3.54 (2.29, 5.46)** | 6.77 (4.29, 10.68)** |
| **Prospective analyses** |  |  |
| ***18 months*** *(n=882)*^¶^ |  |  |
| Quantity for HFA of snack foods | >1831 to ≤3493 kcal, FFQ | >3493 kcal, FFQ |
| Low (≤8610 kcal) | 1.00 | 1.00 |
| Medium (>8610 to ≤11460 kcal) | 1.52 (1.03, 2.26)* | 2.04 (1.30, 3.18)** |
| High (>11460 kcal) | 1.22 (0.80, 1.86) | 2.27 (1.45, 3.56)** |

*p<0.05, **p<0.01

^†^All multinomial logistic regression models adjusted for the following covariates: child’s sex, child’s age, mother’s baseline age, mother’s education, mother’s baseline employment status, mother’s ethnicity, and household size

^‡^36-month cross-sectional analyses (this section of rows) used HFA and diet data from 36 months

^§^*FFQ* food frequency questionnaire

^¶^Prospective analyses (this section of rows) used HFA data from 18 months and diet data from 36 months

**Supplementary Table 5.** Cross-sectional and prospective associations between home food availability (HFA) of sugar-sweetened beverages (SSBs; quantity) and SSB intake (medium or high vs. low) among children 18 and 36 months of age in Pakistani and White households, Born in Bradford 1000 (BiB1000) study^†^

| **HFA at designated time (Exposure)** | **Categories of SSB intake quantity (Outcome)** | |
| --- | --- | --- |
|  | Medium | High |
| **Cross-sectional analyses** |  |  |
| ***36 months*** *(n=971)*^‡^ |  |  |
| Quantity for HFA of SSBs | >334 to ≤1033 kcal, FFQ | >1033 kcal, FFQ |
| Low (≤230 kcal) | 1.00 | 1.00 |
| Medium (>230 to ≤660 kcal) | 2.26 (1.53, 3.35)** | 1.92 (1.27, 2.91)** |
| High (>600 kcal) | 2.33 (1.52, 3.58)** | 4.11 (2.68, 6.29)** |
| **Prospective analyses** |  |  |
| ***18 months (Prospective)*** *(n=882)*^¶^ |  |  |
| Quantity for HFA of SSBs | >325 to ≤1005 kcal, FFQ | >1005 kcal, FFQ |
| Low (≤230 kcal) | 1.00 | 1.00 |
| Medium (>230 to ≤660 kcal) | 1.08 (0.72, 1.62) | 1.31 (0.86, 1.99) |
| High (>660 kcal) | 1.49 (0.95, 2.33) | 2.46 (1.57, 3.84)** |

*p<0.05, **p<0.01

^†^All multinomial logistic regression models adjusted for the following covariates: child’s sex, child’s age, mother’s baseline age, mother’s education, mother’s baseline employment status, mother’s ethnicity, and household size

^‡^36-month cross-sectional analyses (this section of rows) used HFA and diet data from 36 months

^§^*FFQ* food frequency questionnaire

^¶^Prospective analyses (this section of rows) used HFA data from 18 months and diet data from 36 months

**Supplementary Table 6.** Cross-sectional and prospective associations between home food availability (HFA) of snack foods or sugar-sweetened beverages (SSBs; variety and quantity) and body mass index (BMI) among children at 18 and 36 months of age in Pakistani and White households, Born in Bradford 1000 (BiB1000) study^†^

|  | **Time of BMI Measurement (Outcome)** | | | | | | | |
| --- | --- | --- | --- | --- | --- | --- | --- | --- |
|  | **Cross-sectional:**  **18 months (*n*=921)**^‡^ | |  | **Cross-sectional:**  **36 months (*n=*816)**^§^ | |  | **Prospective:**  **36 months (*n*=743)**^¶^ | |
|  | HFA  cut-points | β (95% CIs) |  | HFA  cut-points | β (95% CIs) |  | HFA  cut-points | β (95% CIs) |
| **Snack foods** |  |  |  |  |  |  |  |  |
| Quantity |  |  |  |  |  |  |  |  |
| Low | ≤8610 kcal | 0.00 |  | ≤9290 kcal | 0.00 |  | ≤8505 kcal | 0.00 |
| Medium | >8610 to  ≤11460 kcal | -0.10 (-0.33, 0.13) |  | >9290 to  ≤12107 kcal | -0.07 (-0.31, 0.18) |  | >8505 to  ≤11402 kcal | -0.14 (-0.40, 0.12) |
| High | >11460 kcal | -0.19 (-0.43, 0.05) |  | >12107 kcal | 0.23 (-0.02, 0.49) |  | >11402 kcal | -0.33 (-0.61, -0.05)* |
| **SSBs** |  |  |  |  |  |  |  |  |
| Quantity |  |  |  |  |  |  |  |  |
| Low | ≤230 kcal | 0.00 |  | ≤230 kcal | 0.00 |  | ≤230 kcal | 0.00 |
| Medium | >230 to  ≤660 kcal | -0.21 (-0.45, 0.02) |  | >230 to  ≤660 kcal | 0.08 (-0.17, 0.33) |  | >230 to  ≤660 kcal | -0.24 (-0.50, 0.02) |
| High | >660 kcal | -0.15 (-0.40, 0.09) |  | >660 kcal | 0.17 (-0.09, 0.43) |  | >660 kcal | -0.17 (-0.44, 0.11) |

*p<0.05, **p<0.01

^†^All multivariable linear regression models adjusted for the following covariates: child’s sex, child’s age, mother’s baseline age, mother’s education, mother’s baseline employment status, mother’s ethnicity, and household size

^‡^18-month cross-sectional analyses (this column) used HFA and BMI data from 18 months

^§^36-month cross-sectional analyses (this column) used HFA and BMI data from 36 months

^¶^Prospective analyses (this column) used HFA data from 18 months and BMI data from 36 months

**Supplementary Table 7.** Prospective association between variety of snack foods and sugar-sweetened beverages (SSBs) consumed at 18 months and their respective intake (medium or high vs. low) at 36 months among children in Pakistani and White households, Born in Bradford 1000 (BiB1000) study^†^

| **Variety of intake at 18 months** | **Variety of intake at 36 months** | |
| --- | --- | --- |
|  | Medium | High |
| **Snacks** | >9 to ≤13 FFQ items^‡^ | >13 FFQ items |
| Low (0-2 FFQ items) | 1.00 | 1.00 |
| Medium (3-5 FFQ items) | 1.77 (1.08, 2.90)* | 2.54 (1.39, 4.62)** |
| High (6-7 FFQ items) | 2.43 (1.35, 4.36)** | 5.01 (2.56, 9.81)** |
| **SSBs** | >2 to ≤4 FFQ items | >4 FFQ items |
| None (0 FFQ items) | 0.64 (0.44, 0.95)* | 0.40 (0.25, 0.66)** |
| Low (1 FFQ items) | 1.00 | 1.00 |
| Medium (2 FFQ items) | 1.04 (0.69, 1.58) | 1.31 (0.83, 2.06) |
| High (3-4 FFQ items) | 1.43 (0.73, 2.81) | 2.33 (1.15, 4.69)* |

*p<0.05, **p<0.01

^†^All multinomial logistic regression models adjusted for the following covariates: child’s sex, child’s age, mother’s baseline age, mother’s education, mother’s baseline employment status, mother’s ethnicity, and household size

^‡^*FFQ* food frequency questionnaire
